# Cardiac-Specific Overexpression of Catalytically Inactive Corin Reduces Edema, Contractile Dysfunction, and Death in Mice with Dilated Cardiomyopathy

**DOI:** 10.3390/ijms21010203

**Published:** 2019-12-27

**Authors:** Ranjana Tripathi, Ryan D. Sullivan, Tai-Hwang M. Fan, Aiilyan K. Houng, Radhika M. Mehta, Guy L. Reed, Inna P. Gladysheva

**Affiliations:** 1 Department of Internal Medicine, University of Arizona College of Medicine—Phoenix, Phoenix, AZ 85004, USA; rtripathi@email.arizona.edu (R.T.); ryansullivan@email.arizona.edu (R.D.S.); radhikammehta@gmail.com (R.M.M.); 2 Department of Internal Medicine, University of Tennessee Health Science Center, Memphis, TN 38163, USA; tfan1@uthsc.edu (T.-H.M.F.); aiilyan.houng@gmail.com (A.K.H.); 3 Departments of Comparative Medicine, University of Tennessee Health Science Center, Memphis, TN 38163, USA

**Keywords:** corin, dilated cardiomyopathy, heart failure, edema

## Abstract

Humans with dilated cardiomyopathy (DCM) and heart failure (HF) develop low levels of corin, a multi-domain, cardiac-selective serine protease involved in natriuretic peptide cleavage and sodium and water regulation. However, experimental restoration of corin levels markedly attenuates HF progression. To determine whether the beneficial effects of corin in HF require catalytic activity, we engineered cardiac overexpression of an enzymatically inactive corin transgene (corin-Tg(i)). On a wild-type (WT) background, corin-Tg(i) had no evident phenotypic effects. However, in a well-established genetic model of DCM, corin-Tg(i)/DCM mice had increased survival (*p* < 0.01 to 0.001) vs. littermate corin-WT/DCM controls. Pleural effusion (*p* < 0.01), lung edema (*p* < 0.05), systemic extracellular free water (*p* < 0.01), and heart weight were decreased (*p* < 0.01) in corin-Tg(i)/DCM vs. corin-WT/DCM mice. Cardiac ejection fraction and fractional shortening improved (*p* < 0.01), while ventricular dilation decreased (*p* < 0.0001) in corin-Tg(i)/DCM mice. Plasma atrial natriuretic peptide, cyclic guanosine monophosphate, and neprilysin were significantly decreased. Cardiac phosphorylated glycogen synthase kinase-3β (pSer9-GSK3β) levels were increased in corin(i)-Tg/DCM mice (*p* < 0.01). In summary, catalytically inactive corin-Tg(i) decreased fluid retention, improved contractile function, decreased HF biomarkers, and diminished cardiac GSK3β activity. Thus, the protective effects of cardiac corin on HF progression and survival in experimental DCM do not require the serine protease activity of the molecule.

## 1. Introduction

Heart failure (HF) is a leading cause of morbidity and mortality that affects an estimated 6.2 million US adults, and the prevalence is predicted to increase to more than 8 million by 2030 [1]. Dilated cardiomyopathy (DCM) is a major cause of symptomatic HF and is the most common reason for heart transplantation [2,3,4]. DCM is characterized by low cardiac output and progressive heart enlargement with reduced ejection fraction (rEF), i.e., a decline in systolic function that leads to overt HFrEF characterized by lung and other types of edema (pleural effusion, ascites, pretibial edema, etc.), breathlessness, low cardiac output, and death [4,5]. Patients at risk for DCM have Stage A HF, but usually progress to Stage B, where there is a decline in heart function; both stages are pre-symptomatic, or prior to the development of symptoms. Subsequently, patients develop symptomatic HF with lung and systemic edema, breathlessness, fatigue (Stages C, D), and death [6,7]. Despite advancements in treatment strategy, nearly 50% of patients die within five years of diagnosis [8]. Hence, there is a critical need to discover mechanisms that regulate the development and progression of DCM and HF that might lead to new treatment and prevention strategies.

**Figure 1:**
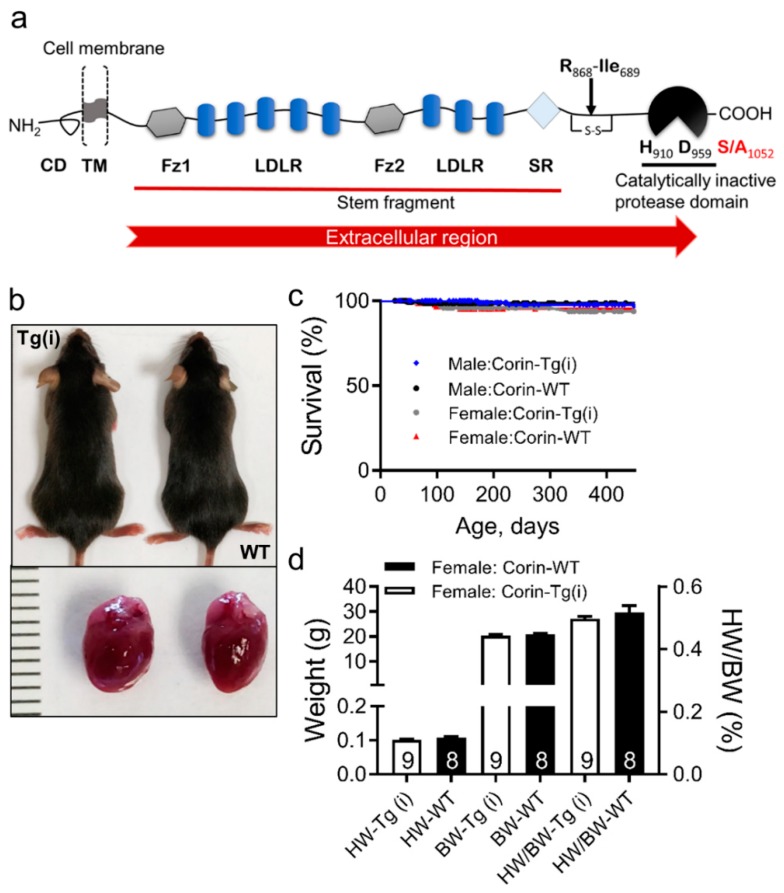
Comparison of corin-Tg(i) and wild-type (WT) control mice. (**a**) Schematic presentation of corin structure with individual domains: cytoplasmic domain (CD), transmembrane domain (TM), frizzled domain1 (Fz1), low-density lipoprotein receptor (LDLR) repeats, frizzled domain2 (Fz2), macrophage scavenger receptor (SR)-like domain, and catalytically inactive protease domain with catalytic triad containing histidine (H_910_), aspartate (D_959_), and serine (S) to alanine (A) mutation at positions 1052 (S/A_1052_) [25,31,32]. (**b**) Representative image of corin-Tg(i) and WT littermate female mice at 90 days (upper panel), and corresponding hearts (lower panel). (**c**) Kaplan–Meier survival curves of female and male corin-Tg(i) vs. sex-matched WT mice (*n* = 125–181 per group). (**d**) Comparison of heart weight (HW), body weight (BW), (left *Y*-axis), and heart weight to body weight ratio (HW/BW) (right *y*-axis) of corin-Tg(i) vs. WT female control mice. Data are represented as mean ± SE.

A growing body of evidence in humans and experimental models suggests that corin, a cardiac transmembrane II serine protease, plays diagnostic, prognostic, and protective roles in DCM and HFrEF development [9,10,11,12,13,14,15,16,17,18,19,20,21,22,23,24]. Cardiac corin is co-expressed with pro-atrial natriuretic peptide (pro-ANP) and localized on the cardiomyocyte surface [25,26], where it cleaves/activates secreted pro-ANP generating biologically active ANP peptides. The catalytic or proteolytic activity of corin is attributed to its C-terminal trypsin-like serine protease domain. Biologically active ANP is released in circulation and binds to the natriuretic peptide-A receptor in kidneys and vasculature [27]. This binding leads to receptor activation and generation of 3′-5′-cyclic guanosine monophosphate (cGMP) and directly inhibits renin release from the kidneys promoting natriuresis, diuresis, and vasodilation [28,29,30]. In addition to the serine protease domain, the extracellular region (ectodomain) of human, mouse, or rat corin is composed of two frizzled-like domains, eight low-density lipoprotein receptor (LDLR) repeats, and a scavenger receptor–like domain (Figure 1a) [25,31,32]. Similar to other transmembrane proteases, the shedding of the corin ectodomain leads to a soluble form of corin circulating in plasma [11,33].

Collectively, experimental and clinical studies have attributed the protective effect of cardiac corin in DCM/HFrEF to the biological activity of its protease domain, which cleaves pro-ANP (and possibly pro-BNP) to biologically active hormones. We reported that plasma corin levels are robustly reduced in patients with acute HFrEF leading to impaired pro-ANP activation, suggesting that reduced corin levels may contribute, in some individuals, to the sodium and water retention associated with HFrEF [11,14,15,19]. Our recent clinical and experimental published data show that corin cardiac expression and plasma levels may reflect the severity of cardiomyopathy, impaired systolic function, and may precede the development of HFrEF [22,23]. Importantly, we found that cardiac-specific expression of catalytically active corin delays the onset of symptomatic HFrEF associated with lung edema and extends life in experimental mouse model of DCM [16,17]. In agreement with our results, corin cardiac transcript levels were upregulated in heart transplant recipients with normal left ventricular (LV) function in comparison with DCM/HFrEF patients [24]. These results suggested that preservation or restoration of cardiac corin levels might delay the progression of HFrEF.

Corin is localized on the cardiomyocyte surface in catalytically active and in catalytically inactive forms [26]. Some of the protective effects of corin in DCM may be mediated through the non-catalytic domains of the molecule. To address this hypothesis, we investigated the effect of transgenic overexpression of catalytically inactive corin (corin-Tg(i)) in the heart of mice with DCM, which develop Stages A-D of human HFrEF in the setting of preserved kidney function [16,22,34,35,36,37,38]. Our results indicate that catalytically inactive corin reduces lung and systemic edema, improves cardiac systolic dysfunction, and prolongs survival.

## 2. Results

### 2.1. Effects of Overexpression of Catalytically Inactive Cardiac Corin-Tg(i) onWild-Type Mice

To determine the effect of overexpression of catalytically inactive corin, we compared corin-Tg(i) mice to wild-type, congenic littermate mice. Corin-Tg(i) mice were viable, fertile, and indistinguishable from C57BL/6J wild-type (WT) littermate mice in appearance and survival (in both sexes) (Figure 1b,c). Corin-Tg(i) mice were similar in body weight and heart weight to the WT mice (Figure 1d) at 90 days of age. Monitoring these mice until 450 days of age revealed no differences in body weights (BW) and heart weights (HW) of male or female corin-Tg(i) vs. sex-matched WT mice (BW; male: 33.0 ± 2.3 vs. 34.1 ± 1.1 g; female: 25.9 ± 1.1 vs. 29.2 ± 1.8 g) and (HW; male: 0.16 ± 0.01 vs. 0.16 ± 0.01 g; female: 0.13 ± 0.00 vs. 0.14 ± 0.01 g).

### 2.2. Catalytically Inactive Cardiac Corin-Tg(i) Improves Survival in Mice with DCM

**Figure 2:**
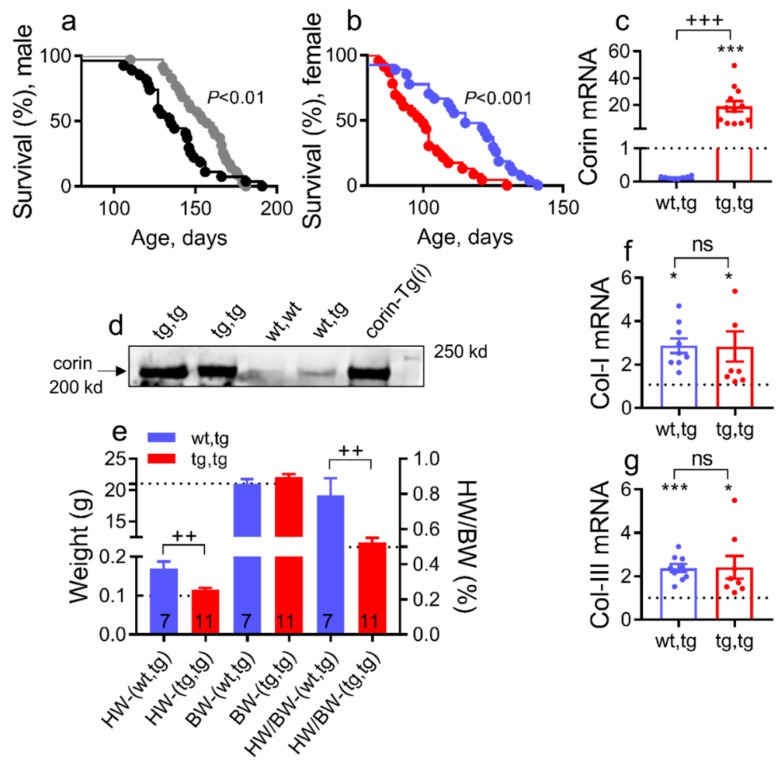
Corin-Tg(i) cardiac overexpression increases survival, reduces heart weights, and does not modulate collagen-I and III cardiac expression in mice with DCM. (**a**,**b**) Kaplan–Meier survival curves of male mice (**a**) with genotype tg,tg (●, *n* = 34) or wt,tg (●, *n* = 27), and female mice (**b**) with genotype tg,tg (●, *n* = 34) or wt,tg (●, *n* = 33). Corin-Tg(i)/DCM= tg,tg and corin-WT/DCM= wt,tg. (**c**) Cardiac expression of corin-Tg(i) transcripts in tg,tg vs. wt,tg mice at 90 days determined by qRT-PCR (*n* = 8–12 per group). (**d**) Corin cardiac protein expression assessed by Western blot under reduced conditions in tg,tg, wt,tg, wt,wt, and corin-Tg(i) female mice groups, 49 days old mice, 50 µg protein per lane [26,39]. (**e**) Comparison of heart weight (HW), body weight (BW) (left *Y*-axis), and heart weight to body weight ratio (HW/BW) (right *y*-axis) in tg,tg vs. wt,tg female mouse groups, at 90 days of age; the number of animals per group is shown. (**f**) Cardiac expression of collagen-I (Col-I) transcripts and (**g**) collagen-III (Col-III) transcripts levels in female tg,tg vs. wt,tg mouse groups (*n* = 8–9 per group). Statistical differences for (**c**,**f**) were analyzed by one-way ANOVA using Newman–Keuls multiple comparison test, and for (**g**) by using Kruskal–Wallis test using Dunn’s multiple comparison test. Differences between two groups (**e**) were analyzed by Student’s *t* test. Dotted lines represent WT levels as a reference. Data are represented as mean ± SE; *** *p* < 0.001, * *p* < 0.05 (tg,tg or wt,tg vs. WT); ^+++^ *p* < 0.001, ^++^ *p* < 0.01 (tg,tg vs. wt,tg); ns = not significant.

DCM mice recapitulate all stages (A–D) of human HF development [22,36]. To examine whether proteolytic activity of corin is required to protect against DCM progression and HF development, we backcrossed DCM mice with mice that selectively overexpress catalytically inactive corin-Tg(i) (Figure 1a) on the same strain background. Survival was monitored in littermates of both sexes—corin-Tg(i)/DCM (tg,tg) and corin-WT/DCM (wt,tg). Male and female corin-Tg(i)/DCM mice survived significantly longer than sex-matched corin-WT/DCM mice (median survival: male, 161 vs. 135 days, Figure 2a, *p* < 0.01 and female, 116 vs. 100 days, Figure 2b, *p* < 0.0001).

Additional studies were performed to better understand the accelerated HF development and mortality in female vs. male mice with DCM [22,36]. Cardiac corin mRNA expression was increased nearly 20-fold in corin-Tg(i)/DCM vs. corin-WT/DCM 90 days old female mice, determined by quantitative real-time polymerase chain reaction (qRT-PCR; Figure 2c, *p* < 0.01). As expected, cardiac corin protein expression was significantly increased in corin-Tg(i)/DCM vs. corin-WT/DCM female mice (Figure 2d). Corin-Tg(i) overexpression significantly reduced heart weight and heart weight to body weight ratio (Figure 2e, *p* < 0.01) in female mice with DCM; no significant changes in BW were observed (Figure 2e). Cardiac transcript levels for collagen-I (Figure 2f) and collagen-III (Figure 2g) were not statistically different between the corin-Tg(i), DCM and corin-WT/DCM groups at 90 days of age, although their levels in both groups were significantly elevated above levels observed in WT mice of similar ages.

### 2.3. Corin-Tg(i) Overexpression Reduces Pleural Effusion, Lung Edema, and Systemic Water Retention in Mice with DCM

**Figure 3:**
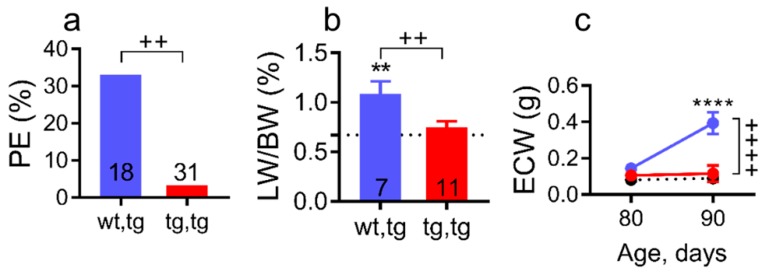
Cardiac corin-Tg(i) overexpression reduces pleural effusion, edema, and systemic water retention in female mice with DCM. (**a**) Pleural effusions (PE) prevalence; bars represent percent affected mice analyzed by Fishers exact test and (**b**) lung weight-to-body weight ratio (LW/BW) at 90 days of age, analyzed by one-way ANOVA with Newman–Keuls multiple comparison test. The number of mice per group is shown and the values for control mice without DCM (corin-WT/WT, wt,wt) are indicated by dotted lines (*n* = 8). (**c**) Age-related changes in extracellular water (ECW). Experimental groups were: Corin-Tg(i)/DCM (●, tg,tg, *n* = 6–11), corin-WT/DCM (●, wt,tg, *n* = 20–25), and WT (dotted lines, *n* = 15–18) mice. Data are represented as mean ± SE; **** *p* < 0.0001, ** *p* < 0.01 (tg,tg or wt,tg vs. wt,wt); ^++++^ *p* < 0.0001, ^++^ *p* < 0.01 (tg,tg vs. wt,tg).

Pleural effusion, lung water retention, or lung edema are clinical manifestations of advanced HF (Stages C–D HF) in human [6,40] and in DCM mice, as we previously reported [22,35,36]. Necropsy analysis of sub-groups of mice at 90 days of age confirmed the presence of pleural effusion and lung edema in corin-WT/DCM mice (Figure 3a,b). Pleural effusion was evident by presence of the pleural fluid in the thoracic cavity and lung edema was assessed by lung weight to body weight ratio (LW/BW, %). Pleural effusion prevalence was significantly decreased in corin-Tg(i)/DCM vs. corin-WT/DCM mice (Figure 3a, 3.3 vs. 33%, *p* < 0.01). Similarly, lung edema (LW/BW) was significantly reduced in corin-Tg(i)/DCM vs. corin-WT/DCM mice (Figure 3b, *p* < 0.05) but was not significantly different from WT group (Figure 3b). Female DCM mice at Stages C-D HF, like humans, accumulate edema in the lungs, and peripheral tissues (systemic edema), which can be measured by as increases in extracellular water (ECW; Figure 3c) using noninvasive quantitative magnetic resonance (QMR) for body composition monitoring as we previously reported [35]. By 90 days of age, corin-WT/DCM mice accumulated significantly elevated ECW levels as compared with corin-Tg(i)/DCM littermate mice (*p* < 0.0001, Figure 3c), though BW (Figure 2e) were relatively comparable. Moreover, corin-Tg(i)/DCM mice maintained normal ECW levels, which were comparable with WT mice (Figure 3c).

### 2.4. Corin-Tg(i) Overexpression Reduces Heart Systolic Dysfunction and Ventricular Dilation in DCM

**Figure 4:**
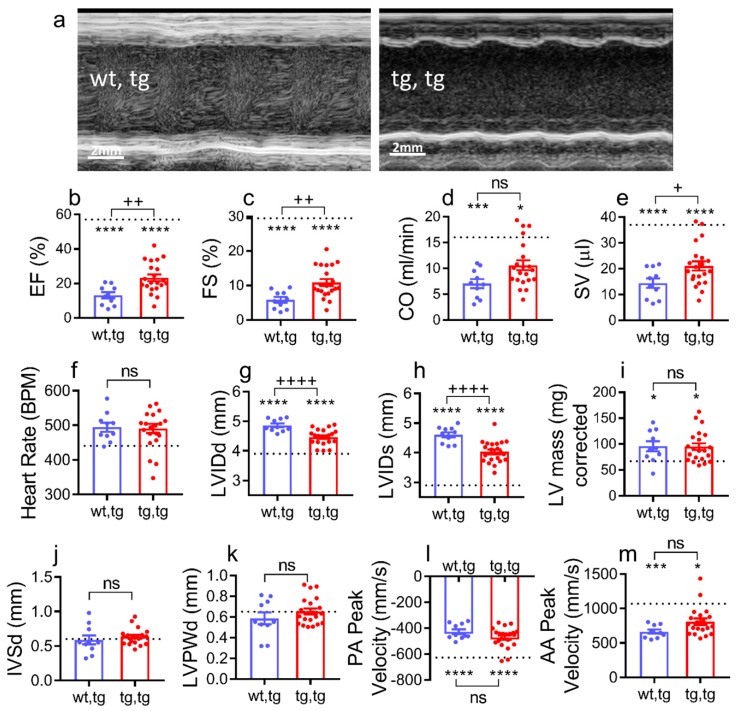
Cardiac corin-Tg(i) overexpression improves systolic function in female mice with DCM. (**a**) Representative two-dimensional guided left ventricular short axis M-mode images, (**b**) ejection fraction (EF), (**c**) fraction shortening (FS), (**d**) cardiac output (CO), (**e**) stroke volume (SV), (**f**) heart rate (beats per minute), (**g**) left ventricular internal diameter in diastole (LVIDd), (**h**) left ventricular internal diameter in systole (LVIDs), (**i**) LV mass, (**j**) interventricular septal wall thickness in diastole (IVSd), (**k**) left ventricular posterior wall thickness in diastole (LVPWd), (**l**) pulmonary artery (PA) peak velocity, and (**m**) ascending aorta (AA) peak velocity in all experimental groups. Experimental groups were mice at 90 days of age: Corin-Tg(i)/DCM (●, tg,tg, *n* = 21) and corin-WT/DCM (●, wt,tg, *n* = 10), and dotted line represents normal control values in corin-WT/WT (wt,wt, *n* = 7). Statistical differences in all panels among three groups were analyzed by one-way ANOVA using Newman–Keuls multiple comparisons test except in (**d**,**i**,**k**,**m**) where Kruskal–Wallis test using Dunn’s multiple comparisons test were used. Data are represented as mean ± SE; **** *p* < 0.0001, *** *p* < 0.001, * *p* < 0.05 (tg,tg or wt,tg vs. wt,wt); ^++++^ *p* < 0.0001, ^++^ *p* < 0.01, ^+^ *p* < 0.05 (tg,tg vs. wt,tg); ns = not significant.

Heart systolic function or contractile function and left ventricular dimensions were evaluated in all experimental groups at 90 days of age by transthoracic echocardiography. Systolic function was better preserved in corin-Tg(i)/ DCM mice, as shown in Figure 4a–c. Representative M-mode images of individual mice from each group showed greater ventricular dilation during systole and diastole in mice with DCM, which was rescued by cardiac corin-Tg(i) overexpression in corin-Tg(i)/DCM mice (Figure 4a). Systolic function, as assessed by ejection fraction (EF, %), and fraction shortening (FS, %) was significantly better in corin-Tg(i)/DCM mice (Figure 4b,c; *p* < 0.01 for both). Cardiac output (CO, mL/min) was positively modulated in corin-Tg(i)/DCM vs. corin-WT/DCM mice (Figure 4d; *p* < 0.05). This was directly related to improvement in stroke volume (SV, µL; Figure 4e), rather than a change in heart rate (BPM; Figure 4f) between groups.

Cardiac dilation was less severe in corin-Tg(i)/DCM vs. corin –WT/DCM mice. Left ventricular internal diameter, diastole (LVIDd, mm; *p* < 0.001, Figure 4g), and LVID systole (LVIDs, mm; *p* < 0.001 Figure 4h) were significantly improved. The left ventricular mass and wall thickness were not statistically different between all three groups as assessed by LV mass corrected, interventricular septum, diastole (IVSd), LV posterior wall, and diastole (LVPWd) (Figure 4i–k).

The pulmonary artery (PA) peak velocity and the ascending aortic (AA) peak velocity were not significantly different between corin-Tg(i)/DCM vs. corin –WT/DCM mice (Figure 4l,m).

### 2.5. Effect of Cardiac Corin-Tg(i) Overexpression on HF Plasma Biomarkers

**Figure 5:**
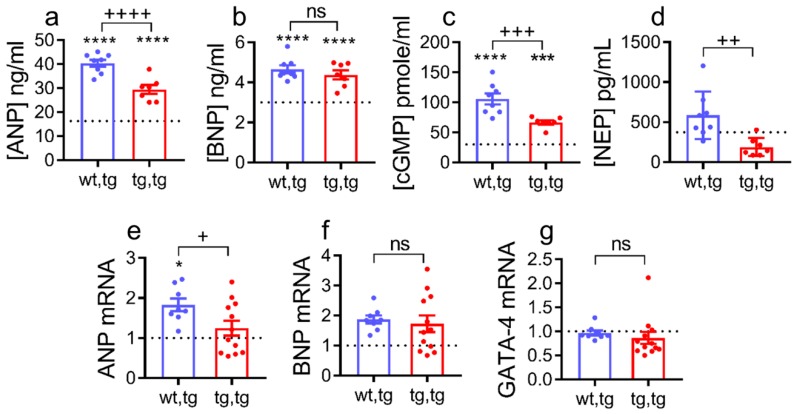
Effect of cardiac corin-Tg(i) overexpression on the natriuretic peptide system plasma biomarkers and cardiac expression levels in female mice with DCM. Plasma levels of (**a**) atrial natriuretic peptide (ANP), (**b**) b-type natriuretic peptide (BNP), (**c**) cyclic guanosine monophosphate (cGMP), and (**d**) neprilysin (NEP), determined by ELISA, *n* = 7–8 per group. Cardiac transcript levels of (**e**) pro-ANP, (**f**) pro-BNP, and (**g**) GATA-4, determined by qRT-PCR, *n* = 8–12 per group. Dotted line represents WT levels (wt,wt; *n* = 6–7). Differences were analyzed by one-way-ANOVA using Newman–Keuls multiple comparison test for (**a**–**f**), and for (**g**), by Kruskal–Wallis test using Dunn’s multiple comparison test. Experimental groups were mice at 90 days of age: Corin-Tg(i)/DCM (●, tg,tg) and corin-WT/DCM (●, wt,tg) mice. Data are represented as mean ± SE; **** *p* < 0.0001, *** *p* < 0.001, * *p* < 0.05 (tg,tg or wt,tg vs. wt,wt); ^++++^ *p* < 0.0001, ^+++^ *p* < 0.001, ^++^ *p* < 0.01, ^+^ *p* < 0.05 (tg,tg vs. wt,tg), ns = non-significant.

Plasma ANP, BNP, cGMP, and neprilysin are well known biomarkers of HF [41] that are elevated in mice with DCM by 90 days of age (Stages C–D HF) [22,35,36]. As expected, these markers were significantly increased above normal WT levels (corin-WT/WT) in both DCM groups (Figure 5). Plasma levels of ANP were significantly decreased in corin-Tg(i)/DCM vs. corin-WT/DCM mice (*p* < 0.0001, Figure 5a), while BNP plasma levels were unchanged between the two DCM groups (Figure 5b). In agreement with this, plasma cGMP levels were significantly lower in corin-Tg(i)/DCM mice (*p* < 0.001, Figure 5c). Cardiac overexpression of corin-Tg(i) suppressed neprilysin plasma levels to the normal physiological levels (*p* < 0.01, Figure 5d).

Similar to plasma levels, cardiac pro-ANP transcript levels were decreased significantly (*p* < 0.01, Figure 5e) in corin-Tg(i)/DCM vs. corin-WT/DCM mice, while cardiac pro-BNP transcript levels were not altered (Figure 5f). Transcript levels of GATA-4, a common transcription factor for corin, ANP, and BNP [32] remained unchanged among all three groups (Figure 5g).

**Figure 6:**
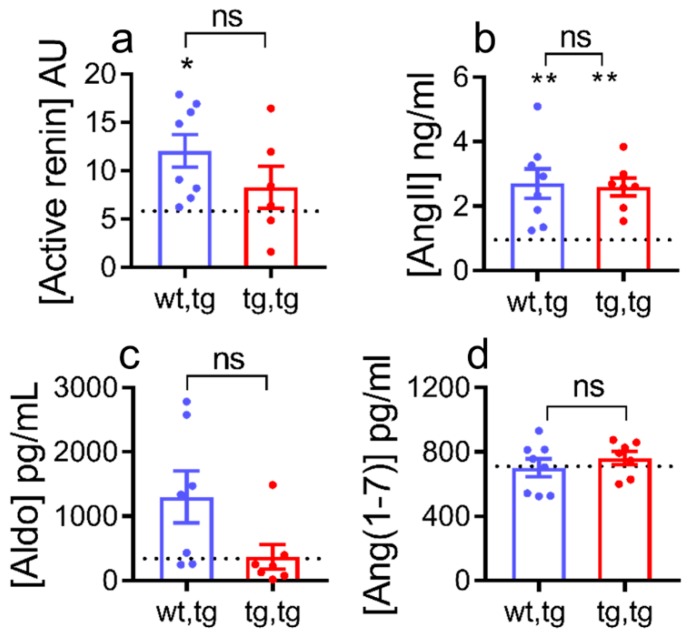
Effect of cardiac corin-Tg(i) overexpression on plasma RAAS biomarkers in female DCM mice. Plasma levels of (**a**) active renin (arbitrary units, AU), (**b**) angiotensin II (AngII), (**c**) aldosterone (Aldo), and (**d**) angiotensin 1–7 (Ang(1–7)) in corin-Tg(i)/DCM (●, tg,tg) and corin-WT/DCM (●, wt,tg) mouse groups, at 90 days of age, *n* = 6–8 per group; dotted line represents control levels in corin-WT/WT (wt,wt) mice. Data are represented as mean ± SE, differences were analyzed (for (a), (b), and (d)) by one-way-ANOVA using Newman–Keuls multiple comparison test, and by Kruskal–Wallis test using Dunn’s multiple comparison test (for (c)). ** *p* < 0.01, * *p* < 0.05 (tg,tg or wt,tg vs. wt,wt); ns = non-significant.

Systemic activation of the renin-angiotensin-aldosterone system (RAAS) occurs in HF with increased plasma renin activity, angiotensin II, and aldosterone levels [42,43,44,45,46,47]. We reported that systemic RAAS is significantly activated in female mice with DCM starting from Stage B HF [35,36]. As expected, plasma renin activity, angiotensin II, and aldosterone levels were significantly elevated in corin-WT/DCM mice compared to normal levels in corin-WT/WT littermates (Figure 6a–c). In corin-Tg(i)/DCM mice, plasma renin activity (*p* = 0.55, Figure 6a) and angiotensin II plasma levels (*p* = 1.0, Figure 6b) were not statistically altered, while aldosterone plasma levels had a non-significant decrease and a trend towards normal levels (*p* = 0.07, Figure 6c). Plasma levels of angiotensin (1–7) were not altered by corin-Tg(i) overexpression (*p* = 0.7, Figure 6d).

### 2.6. Cardiac Corin-Tg(i) Overexpression Increases pSer9-GSK3β Levels in DCM

**Figure 7:**
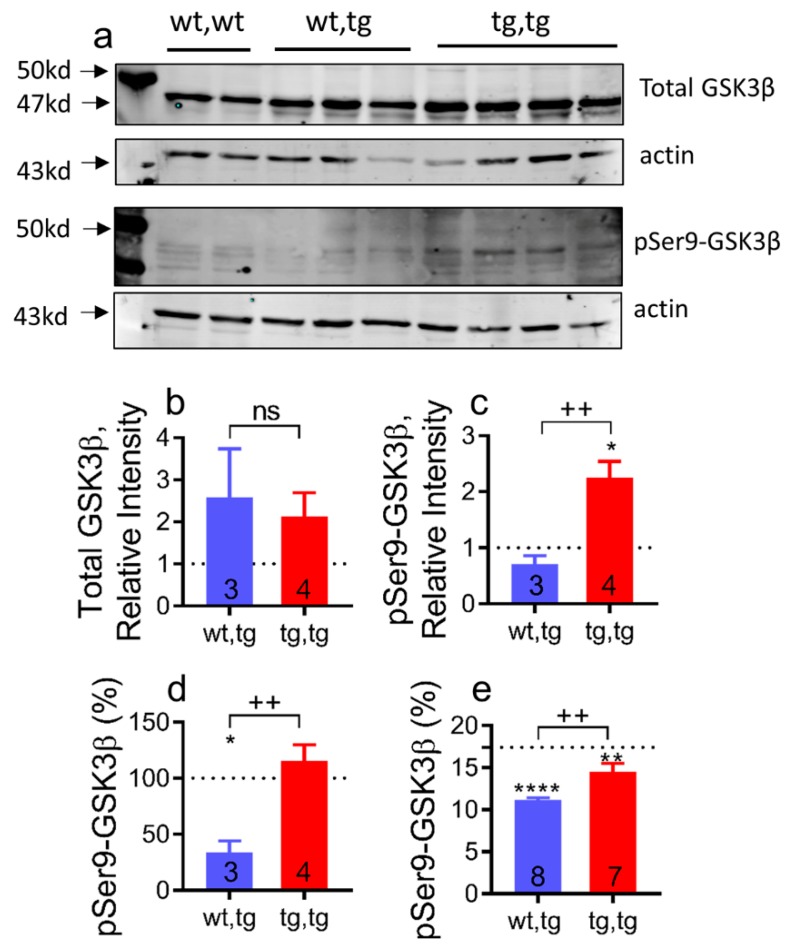
Corin-Tg(i) overexpression modulates pSer9-GSK3β levels in female DCM mice. (**a**) Western blot analysis of cytosol fraction of heart tissue extracts, with antibodies directed against total GSK3β and pSer9-GSK3β under reducing conditions; each lane represents an individual mouse sample. Row 1: Total GSK3β levels (47 kD). Row 2: Actin (43 kD) as loading control for total GSK3β. Row 3: pSer9-GSK3β levels (47 kD) in same set of mice. Row 4: Actin (43 kD) as loading control for pSer9-GSK3β. (**b**–**d**) Bar graphs represent the densitometry analysis of data presented on panel (a) by using Image J software: (**b**) Total GSK3βlevels, (**c**) pSer9-GSK3β levels in corin-WT/DCM (wt,tg) and corin-Tg(i)/DCM (tg,tg) groups as a ratio to corin-WT/WT (wt,wt levels), normalized to corresponding actin levels and represented as relative intensity. (**d**) pSer9-GSK3β (%) levels represented as a ratio to total GSK3β levels; (**e**) ELISA analysis of cytosol fraction of heart tissue extracts, with antibodies directed against total GSK3β and pSer9-GSK3β. pSer9-GSK3β (%) levels represented as a ratio to total GSK3β levels. Dotted line represents corin-WT/WT (wt,wt; *n* = 7) mice. The number of mice per group is shown. Differences were analyzed by one-way-ANOVA using Newman–Keuls multiple comparison test. Data are represented as mean ± SE; **** *p* < 0.0001, ** *p* < 0.01, * *p* < 0.05 (tg,tg or wt,tg vs. wt,wt); ^++^ *p* < 0.01 (tg,tg vs. wt,tg); ns = non-significant.

Abnormal glycogen synthase kinase 3β isoform (GSK3β) activity is implicated in the pathogenesis of HF and in cardiac cell survival pathways that may interact with the LDLR, frizzled, and scavenger structural domains (i.e., non-catalytic domains) of corin (Figure 1a) [48,49,50]. To evaluate the effect of cardiac overexpression of catalytically inactive corin^S1052A^ mutation on cardiac GSK3β expression and activity, we determined total GSK3β protein expression levels and levels of GSK3β phosphorylated at Ser9 (pSer9-GSK3β) in the cardiac cytosol fraction by Western blot analysis and enzyme-linked immunoassays (ELISA). Total GSK3β protein levels were increased in both corin-Tg(i)/DCM and corin-WT/DCM experimental groups vs. corin-WT/WT group (Figure 7a,b). Levels of pSer9-GSK3β were comparable between corin-WT/DCM and corin-WT/WT groups. Overexpression of corin-Tg(i) caused more than a 2-fold upregulation (*p* < 0.01) in the pSer9-GSK3β protein expression levels above corin-WT/WT level or corin-WT/DCM group (Figure 7a,c). Respectively, the relative expression of pSer9-GSK3β, % normalized to total GSK3β protein level, was more than 2-fold upregulated (*p* < 0.01) in corin-Tg(i)/DCM vs. corin-WT/DCM mice (Figure 7d). Consistently, in ELISA assays, the relative expression of pSer9-GSK3β, normalized to total GSK3β protein levels, were significantly increased (*p* < 0.01) in heart tissue extracts of corin-Tg(i)/DCM vs. corin-WT/DCM mice groups approaching comparable levels to corin-WT/WT littermates (Figure 7e).

## 3. Discussion

Converging lines of evidence from clinical and translational studies have shown that genetic abnormalities and reduction in corin cardiac expression level negatively affect DCM and HFrEF outcomes. Cardiac transcripts and circulating levels of corin are depressed in patients and translational animal models with DCM and HFrEF [9,10,11,12,13,14,15,16,17,18,19,20,21,22,23,24,35,36,51], and are pathologically modulated in DCM before the onset of edema and other clinical signs and symptoms of HFrEF [22,23]. In a translationally relevant mouse model of DCM-HFrEF with reduced cardiac and plasma corin levels, we reported that genetic restoration of cardiac-specific catalytically active, native corin improved systolic function, reduced myocardial fibrosis, decreased edema, and prolonged survival [16,17]. Yet, it remains unknown whether catalytic/protease activity of corin is essential for its protective effects in DCM-HFrEF or whether other domains of multi-domain corin structure contribute. In the current study, we used a well-established experimental model of DCM-HFrEF in mice [16,22,34,35,36,37,38] to determine if HF progression would be attenuated by the cardiac-specific, transgenic overexpression of catalytically inactive corin, which carries an inactivating mutation in the catalytic triad of the serine protease domain (corin-Tg(i)) (Figure 1a). Genetic overexpression of corin-Tg(i) did not alter the phenotype and life span of wild-type mice of both sexes. However, when expressed in DCM mice, corin-Tg(i) significantly improved LV systolic function, delayed the development of pleural effusions, pulmonary, and peripheral edema. It reduced plasma levels of ANP, cGMP, and neprilysin, and significantly prolonged life in male (by 19%) and female mice (by 16%).

Overexpression of inactive corin-Tg(i) in DCM mice led to a delayed progression of HFrEF. There were improvements in contractile function of the heart as measured by EF and FS. The heart pumping efficiency, assessed by cardiac output and stroke volume, was significantly improved. LV dilation (LVID) was also significantly improved [16,22,34,35,36,37,38]. No changes in wall thickness and overall cardiac mass were found.

The pathophysiological effects of transgenic overexpression of catalytically active [16] and inactive corin (this report) in DCM mice were similar with important exceptions. Cardiac-specific overexpression of catalytically active corin down-regulated the expression of collagen-I and collagen-III cardiac transcripts, associated with myocardial fibrosis in DCM mice [16]. In contrast, catalytically inactive corin did not modulate expression level of these transcripts. In a similar fashion, cardiac pro-ANP transcript expression and plasma cGMP (second messenger mediating ANP actions) levels in DCM mice were enhanced by restoration of catalytically active corin [16], but depressed by restoration of catalytically inactive corin levels. These data suggest that enzymatic activity of cardiac corin alters ANP levels and exerts anti-fibrotic effects in experimental HFrEF.

Overexpression of corin-Tg(i) in DCM mice delays development of Stages C-D HF as objectively assessed by lung and systemic edema, undetectable pleural effusion levels, and reduced HF plasma biomarker levels: ANP, cGMP, neprilysin, and aldosterone. Most recently, reduced edema or congestion was independently associated with improved quality of life and prognosis, including mortality, in patients with HFrEF treated with angiotensin receptor-neprilysin inhibitor [52]. Attenuation of edema and pleural effusion in corin-Tg(i)/DCM mice may be associated with less volume and pressure overload and improved contractile function as a compensatory effect [53]. Reduced edema is also associated with normalization of circulating neprilysin level as reported for patients with HFrEF and in experimental HF [35,52,54] and trend toward down-regulation of renin plasma activity and aldosterone levels [44,47,55,56].

HF continues to increase in incidence and prevalence [1]. To understand the pathophysiological and molecular basis of DCM-HFrEF progression and to obtain translationally relevant discoveries, it is important to have pre-clinical animal models that recapitulate the stages and biomarker hallmarks of human HF. While no model fully mimics human disease, the mouse model of DCM-HFrEF used in this study has translational relevance to human HFrEF. It exhibits pathological features of clinical HF including edema and cachexia/sarcopenia in the setting of preserved renal function. It has a well-defined plasma biomarker and symptomatic profile, it is responsive to known therapies, and is compliant with recommendations of the AHA Scientific Statement for preclinical animal models of HF [16,22,34,35,36,37,38,57].

Although the molecular mechanisms responsible for the protective effects of catalytically inactive corin in experimental HF are not defined, these studies provide important clues and hypotheses. Cardiac-specific overexpression of corin-Tg(i) upregulated pSer9-GSK3β/total GSK3β levels toward corin-WT/WT controls. Phosphorylated GSK3β myocardial levels are involved in regulation of protein synthesis pathways associated with cachexia [58,59] and cardiac Wnt signaling activation [50,60]. The upregulated pSer9-GSK3β/total GSK3β levels we observed may be the result of Wnt–corin interactions or it may be the result of the overall improvement in systolic function observed in these mice. Wnt activation promotes HF by triggering developmental reprogramming in the adult heart [61]. The interaction of Wnt proteins with their receptors on the cell surface is the first step in signal transduction mechanism [50,62]. The extracellular domain of corin contains two frizzled and eight LDLR domains (Figure 1a) [25,30,31,32], which might serve as alternative/comparative receptor(s) for Wnt, attenuating or enhancing signaling. Further studies will be needed to determine whether the protective effects of the non-catalytically active form of corin on HF progression and survival are mediated through Wnt or other interactions.

## 4. Materials and Methods

### 4.1. Institution and Environment

All experimental animal studies were reviewed and approved by the Institutional Animal Care and Use Committees at the University of Tennessee Health Science Center (15-050; approved 9 July 2015 and 17-059; approved 26 July 2017) and the University of Arizona College of Medicine, Phoenix (17-303; approved 11 December 2017), and were conducted within AAALACi accredited facilities in accordance with the National Institutes of Health (NIH) Guide for the Care and Use of Laboratory Animals.

### 4.2. Mice

The DCM mouse model expresses a dominant negative CREBS133A transcription factor that is targeted to cardiomyocytes via the alpha myosin heavy chain promoter; it has been extensively characterized previously [16,22,34,35,36,37,38]. DCM mice develop progressive systolic dysfunction with transition to symptomatic HFrEF and early death in the setting of preserved kidney function [16,22,34,35,36,37,38]; they are compliant with the AHA Scientific Statement requirements for preclinical animal models of HF [57]. DCM mice are responsive to pharmacological and genetic treatments, which modulate edema, systolic function, progression of cardiomyopathy, and survival [16,35,37]. Mice that overexpress an enzymatically inactive corin transgene contain a single mutation in serine protease catalytic triad, serine 1052 to alanine (S1052A) (corin-Tg(i)); this transgene is driven by the alpha myosin heavy chain promoter as previously described [16,34,63]. Corin-Tg(i)/DCM (tg,tg), corin-WT/DCM (wt,tg), and corin-WT/WT (wt,wt) mice were generated by extensively backcrossing corin-Tg(i) mice with DCM mice, on C57BL/6J background. Mice were housed under a 12:12 light–dark cycle, in the same racks of the individually ventilated caging system (Optimouse, Animal Care Systems; Centennial, CO, USA); and fed an ad-lib maintenance diet (Envigo Teklad 7912; Madison, WI, USA). Investigators and animal care staff monitored the colony daily for health and behavioral changes. All analysis and health/death reports were recorded by investigators and animal facility technicians blinded to the mouse genotype. Littermate male and female mice were used for survival studies. Littermate female mice develop HF at an earlier age than males [36].

In sub-groups of 90-day mice, terminal blood was collected via cardiocentesis with ethylenediaminetetraacetic acid (EDTA)-aprotinin syringes to prevent coagulation and proteolysis; organs were dissected, weighed, and snap frozen in liquid nitrogen. Aliquoted plasma samples and organs were stored at −80 °C until analysis.

### 4.3. Quantitative Real-Time Polymerase Chain Reaction (qRT-PCR)

Total RNA was extracted from snap-frozen heart tissue using RNeasy^®^ Mini Kit (Qiagen, Hilden, Germany). First-strand complementary DNA synthesis was performed with 1 µg of total RNA (Transcriptor First Strand cDNA Synthesis Kit, Roche, Basel, Switzerland). Quantitative real-time PCR (qRT-PCR) was performed using the LightCycler^®^ 480 System following the manufacturer’s protocol as we described [22,37]. Specific primers were: ctggaaggattgctttggag and acgctcctgtctgctctca for corin; tccatcagaggggtcacac and gccttgtgaaggggtgatta for BNP; cacagatctgatggatttcaaga and cctcatcttctaccggcatc for ANP; catgttcagctttgtggacct and gcagctgacttcagggatgt for collagen-I; gcagctgacttcagggatgt and tgagtcgaattggggagaat for collagen-III; and ggaagacaccccaatctcg and catggccccacaattgac for GATA-4. PCR was performed at 95 °C for 5 min, followed by 40 cycles of 95 °C (10 s), 60 °C (30 s), and 72 °C (10 s). PCR products were confirmed by melting curve analysis using the Lightcycler Software 4.0 and samples normalized to a β-actin control [22]. Experiments were performed in triplicate.

### 4.4. Echocardiography

Transthoracic echocardiograms were performed using VisualSonic Vevo 2100 Imaging System (VisualSonic Inc. Toronto, Canada) as we previously described [16,22,35,36,37]. Fur from the ventral thorax was removed by chemical depilatory cream (Nair, Chuch and Dwight Co, Inc. Ewing, NJ, USA) one day before the echocardiographic studies. Briefly, 90-day-old female mice were anesthetized with 1.5% inhaled isoflurane with oxygen. Two-dimensional and M-mode images of the heart and vasculature were obtained from the parasternal long-axis and short axis acoustic windows. Analysis was blindly completed post-recording using Vevo LAB software (3.1.0, VisualSonics) with three cardiac cycles traced to produce mean values. Heart function and morphometrics were measured directly or calculated using standard equations within the software. Pulse wave Doppler was used to access aortic and pulmonary artery velocities.

### 4.5. Lung Edema and Pleural Effusion Analysis

Lung edema was assessed by lung weight/body weight ratios (LW/BW, %) as described before [22,35,36,37]. In brief, right and left lungs were excised and immediately weighed. Then, the LW/BW ratio was calculated as right plus left lung weight divided by BW. Pleural effusion (PE) was accessed by necropsy analysis of the mouse thoracic cavity and it was evident by visual examination: Thoracic cavity and lungs were immersed in the pleural fluid [22,36]. Quantification of PE was done by accessing the prevalence in particular groups of mice and a graph was plotted as percent of PE prevalence in corin-Tg(i)/DCM vs. corin-WT/DCM mice.

### 4.6. Body Composition

Systemic extracellular water (ECW) was objectively recorded and quantified using quantitative magnetic resonance (QMR) technology with EchoMRI^™^ 4-in-1 Analyzer (Echo Medical Systems, Houston, TX, USA) as we have described [35]. Data for some mice in DCM and WT groups have been reported [35]. Mice were fully conscious and minimally restrained throughout each 90 s recording and were returned to their home enclosure following measurement.

### 4.7. Mouse Heart Tissue Extraction

All tissue extraction procedures were performed at 4 °C. All buffers were supplemented with protease inhibitor cocktail and EDTA (Roche, Basel, Switzerland). Frozen hearts were homogenized in ice-cold 10mM Tris–HCl, pH 7.4 buffer and centrifuged at 10,000× *g* for 30 min. The supernatant (cytosol fraction) was collected, aliquoted, stored at −80 °C, and used for analysis of GSK3β and pSer9-GSK3β levels by Western blot and ELISA. For whole tissue extraction, frozen hearts were homogenized in 1X RIPA buffer, 1.5% SDS, and incubated for 2 h. The homogenates were centrifuged at 10,000 rpm for 30 min and supernatant was collected, aliquoted, stored at −80 °C, and used for Western blot analysis of cardiac expressed corin. The protein concentration in extracted samples were determined by the Protein Assay kit (Pierce Biotechnology, Waltham, MA, USA).

### 4.8. Western Blot Analysis

Western blot analysis was performed as described previously [26]. Briefly, equivalent amounts of extracted proteins were subjected to SDS-PAGE under reduced conditions and electro-blotted to Immobilon-P polyvinylidene fluoride (PVDF) membrane (Millipore Corp., Bedford, MA, USA). Blotted PVDF membrane was blocked with 1% BSA, probed with rabbit polyclonal anti-mouse corin stem domain antibody as described [26,39] or rabbit monoclonal anti-GSK3β/anti-pSer9GSK3β (Cell Signaling Technology, Inc., Danvers, MA, USA), followed by incubation with goat anti-rabbit secondary antibody (Licor, Lincoln, NE, USA) and scan by Odyssey Imaging System (Li-COR Bioscience, Lincoln, NE, USA), using image studio software. The membrane was further stripped with stripping buffer (Thermo Fisher Scientific, WA, USA), blocked with 1% BSA, and stained with goat anti-actin antibody (Santa Cruz Biotechnology, Inc., Dallas, TX, USA) to show protein loading. Total GSK3β or pSer9GSK3β levels were calculated in Western blot images by using Image J software (NIH software, Bethesda, MD, USA) and bar graphs were plotted as total GSK3β or pSer9-GSK3β levels in corin-WT/DCM (wt,tg) and corin-Tg(i)/DCM (tg,tg) mice relative to corin-WT/WT (wt,wt) mice, normalized to actin loading control and represented as relative intensity. Further, pSer9-GSK3β (%) levels were calculated as pSer9-GSK3β levels divided by total GSK3β levels and multiplied by 100.

### 4.9. Enzyme Immunoassay

Plasma ANP (as N-terminus- ANP), BNP (as C-terminus- BNP), cGMP, neprilysin, Ang II, and aldosterone levels were measured by ELISA according to manufacturer’s protocol (Phoenix Pharmaceuticals Inc., Burlingame, CA, USA; Enzo Life Science Inc., Farmingdale, NY, USA; Boster Biological Technology, Pleasanton, CA, USA; Abcam Inc., Cambridge, MA, USA) as described before [22,35,36]. Plasma Ang (1–7) levels were measured by ELISA according to manufacture protocol (Wuhan Fine Biotech Co., Ltd., China). Total GSK3β and pSer9-GSK3β levels in equivalent amounts of extracted mouse heart proteins (cytosol fraction) were measured by ELISA according to manufacture protocol (RayBiotech., Inc., Peachtree Corners, GA, USA), and pSer9-GSK3β (%) levels were calculated as pSer9-GSK3β levels divided by total GSK3β levels and multiplied by 100 and plotted.

### 4.10. Plasma Renin Activity Assay

Renin enzymatic activities in plasma samples were measured and quantified by cleavage of exogenous fluorescence resonance transfer (FRET) peptide substrates of renin FRET-QXL™520/5-FAM, optimized for mouse renin using a SensoLyte 520 mouse renin assay kit (AnaSpec, Fremont, CA, USA) as previously reported [35,36,37,47].

### 4.11. Statistical Analysis

Statistical analysis was performed with Graph Pad Prism software 7.0 (GraphPad Software, La Jolla, CA, USA). Survival was analyzed by the Kaplan–Meier method and the comparison of two groups was assessed by the Mantel–Cox test. Age-related differences among various genotype groups in extracellular water, fat, and lean mass were analyzed by Two-way-ANOVA with Bonferroni multiple comparison test. Differences among more than two groups were analyzed by one-way ANOVA with Neumann–Keuls post-hoc correction or non-parametric Kruskal–Wallis test with Dunn’s multiple comparison. Differences between two groups were analyzed by Student’s *t*-test. Categorical data (PE) were analyzed by Fisher exact test. Differences were considered to be significant if *p* ≤ 0.05. The number of animals (*n*) are indicated in Figure or Figure legends. Data are expressed as mean ± SEM.

## 5. Conclusions

We evaluated the potential non-catalytic effects of corin on HFrEF progression. Cardiac-specific overexpression of catalytically inactive corin markedly attenuated HFrEF and improved survival in mice with DCM. It reduced fluid retention, cardiac dilation, and slowed the decline in heart systolic function. Catalytically inactive corin decreased plasma biomarkers of HF and modulated cardiac GSK3β activity. This suggests that the non-catalytically active domains in corin play a significant role in the pathophysiology of DCM and HFrEF.

## Abbreviations

wt,tg	corin-WT/DCM,
tg,tg	corin-Tg(i)/DCM
wt,wt	WT/WT
Corin-Tg (i)	corin^S1052A^-Tg
Tg	transgenic
WT	wild-type
HF	heart failure
rEF	reduced ejection fraction
HFrEF	heart failure with reduced ejection fraction
DCM	dilated cardiomyopathy
RAAS	renin-angiotensin-aldosterone-system
NP	natriuretic peptide
ANP	atrial natriuretic peptide
BNP	b-type natriuretic peptide
Ang II	angiotensin II
Aldo	aldosterone
Ang (1–7)	angiotensin (1–7)
NEP	neprilysin
cGMP	cyclic guanosine monophosphate
GSK3β	glycogen synthase kinase 3β
pSer9-GSK3β	glycogen synthase kinase 3β phosphorylated at Ser9
ELISA	enzyme-linked immunosorbent assay
qRT-PCR	quantitative real-time polymerase chain reaction
AU	arbitrary units
SEM	standard error of the mean
CD	cytoplasmic domain
TM	transmembrane
LDLR	low density lipoprotein receptor
Fz	frizzled
SR	macrophage scavenger receptor
H	histidine
D	aspartate
S	serine
A	alanine
BW	body weight
HW/BW	heart weight to bodyweight ratio
LW/BW	lung weight to bodyweight ratio
QMR	quantitative magnetic resonance
ECW	extracellular water
LV	left ventricle
EF	ejection fraction
FS	fraction shortening
CO	cardiac output
SV	stroke volume
BPM	beats per minute
LVIDd	left ventricular internal diameter, diastole
LVIDs	left ventricular internal diameter, systole
LVMc	left ventricular mass corrected
IVSd	interventricular septum, diastole
LVPWd	left ventricular posterior wall, diastole
PA	pulmonary artery
AA	ascending aorta
PE	pleural effusion
